# Supplementary material for: Are carbon emissions trading and green financial instruments synergistic? -Comprehensive quantitative research based on content analysis
Source: PLoS One. 2024 Mar 7;19(3):e0298601. doi: 10.1371/journal.pone.0298601 (PMC10919601; doi:10.1371/journal.pone.0298601)
Supplement: S1 Data — (ZIP) [file pone.0298601.s001.zip › Data/地级市Z_F工作报告.zip!/╕ú╜¿/╚2├≈╩╨/└┤╫╘╣1⁄2╓┌║┼í╛┬φ┐╦╩2╛▌═°í┐_2013.docx]

各位代表：

　　现在，我代表三明市人民政府向大会作政府工作报告，请予审议，并请市政协各位委员和其他列席人员提出意见。

　　一、2013年工作回顾

　　2013年，我市各级政府在省委、省政府和市委的正确领导下，在市人大及其常委会、市政协的监督支持下，坚持以邓小平理论、“三个代表”重要思想、科学发展观为指导，认真学习贯彻党的十八大和十八届二中、三中全会和习近平总书记系列重要讲话精神，把握稳中求进的工作总基调，全力推动科学发展、跨越发展，较好地完成了市十二届人大二次会议确定的目标任务。初步预计，全市生产总值1489.2亿元，增长11.3%；规模以上工业增加值705.5亿元，增长14.7%；公共财政总收入136.9亿元，增长12.5%，其中地方公共财政收入89.8亿元，增长16%；固定资产投资1334.4亿元，增长22.1%；实际利用外商直接投资1.2亿美元，增长12.6%；社会消费品零售总额360.1亿元，增长12.8%；城镇居民人均可支配收入25650元，增长9.5%；农民人均纯收入10546元，增长12.5%；居民消费价格总水平上涨2.4%；节能减排年度任务可以完成。一些重要领域、重点项目取得突破：

　　——建泰高速公路建成通车，实现县县通高速公路；向莆铁路开通运营，南三龙铁路、海西天然气管网三明段一期工程等一批重大基础设施项目动工建设。

　　——三明生态工贸区生态新城建设全面启动，中节能海西（三明）节能环保产业园、福建台明年产20万吨离心球墨铸铁管等一批重大产业项目落地。

　　——沙县创新农村金融改革经验得到国务院领导批示肯定；招商银行三明分行、福建海峡银行三明分行和7家村镇银行新设开业，沙县农村商业银行改制开业。

　　——12个县（市、区）全部列入原中央苏区范围；我市荣获福建省森林城市称号；泰宁县成为全省首批国家生态县，7个县（市）通过省级生态县（市）验收。

　　——我市公立医院改革经验得到国家有关部门和省上肯定；荣获全国综治“长安杯”；在2013年度全国城市文明程度指数测评中位居全省提名资格城市第一；被列入第二批国家公共文化服务体系示范区创建城市；三明籍运动员在第十二届全运会上获得2金3银4铜。

　　一年来的主要工作和成效是：

　　（一）在稳中求进中促进经济稳定增长。面对复杂多变的经济形势，认真落实中央和省上稳增长促发展各项政策，制定出台稳定工业增长13条、扶持外经贸发展、金融服务支持小微企业等政策，助推实体经济加快发展。加大企业帮扶力度，实行“一企一策”，支持企业开拓市场，设立企业应急转贷专项资金和融资担保风险补偿金，帮助企业渡过难关。继续打好“五大战役”，加强重点项目跟踪督查，全年实施重点项目1375个，完成投资1018亿元，占年度计划的121%，790个项目建成投产或投入使用。推进“三维”项目对接，397个项目列入省“三维”系统跟踪管理，全年完成投资319.12亿元。

　　（二）在调整结构中加快产业转型升级。突出抓龙头、铸链条、建集群，制定出台工业产业链拓展延伸实施方案，组织开展352项投资千万元以上重点技改项目，机械科学研究总院海西高端装备产业园等项目加快建设，56家企业被省上认定为战略性新兴产业企业，八大重点产业产值增长14.8%，占全市规模工业总产值的88.7%。制定实施千亿现代农业产业跨越发展行动计划和设施农业发展规划，培育壮大中国利农、清流春舞枝花卉等龙头企业，创建三明茶业公共品牌“尚书品”，市级以上农业龙头企业产值突破300亿元。抓好120个服务业重点项目，现代物流、电子商务等产业加快发展，文化产业增加值增幅保持全省第一，游客接待量、旅游总收入分别增长16.5%和17%，成功申报三明郊野国家地质公园和清流温泉国家地质公园。推动科技创新，在全省率先设立科技型中小企业贷款风险补偿金，市本级及12个县（市、区）通过全国市县科技进步考核，全市高新技术产业产值突破500亿元。

　　（三）在统筹城乡中推动区域协调发展。加强城乡基础设施建设，漳永高速公路、湄渝高速公路莘口至明溪城关段、三明沙县机场加快建设，浦建龙梅、长泉铁路前期工作取得进展，尤溪二期、金溪三期防洪工程等一批重大水利项目开工。加快三明生态工贸区建设，三明中心城市快速通道和205国道、306省道改线工程稳步实施，三明市区与沙县、永安同城化迈出新步伐。大力发展县域经济，推动省级扶贫开发工作重点县发展，“大城关”、小城镇和美丽乡村建设有序推进，“点线面”攻坚持续深化，完成造福工程搬迁和危房改造9550户，少数民族乡村加快发展。扎实推进国家级生态市和国家环保模范城市创建工作，植树造林和“四绿”工程建设任务超额完成，水土流失综合治理取得成效。

　　（四）在改革开放中增添发展动力活力。深化集体林权制度改革，继续保持全国全省领先地位。创新林博会办会方式，建成全国首家林业大宗商品交易中心，开通“林品汇”网上商城，打造“天天林博会”，林业大宗商品交易额突破1500亿元。推进小城镇机构改革试点工作，基本完成事业单位分类工作。深化预算管理制度改革和国库集中支付改革，积极推进公务卡结算制度。汉堂生物、华伦特重工等8家企业在海峡股权交易中心挂牌，市国投公司二期10亿元、市城投公司首期18亿元企业债券获批发行，中小企业私募债券发行实现零的突破。矿产资源整合力度加大，我市被列入国家资源成熟型城市。优化通关环境，生产型外贸企业出口较快增长。支持民营经济发展，民间投资占全市固定资产投资的比重达76%。区域协作不断加强，明港澳台合作进一步深化。

　　（五）在为民利民中保持社会和谐稳定。全面完成省市26项为民办实事项目，全市民生支出比上年增长23.4%，占公共财政支出的比重达77.6%。全年农村富余劳动力转移就业4.77万人，新增城镇就业2.46万人，城镇登记失业率2.11%。社会保险覆盖面逐步扩大，城乡居民社会养老保险全覆盖，实现城乡居民医疗保险一体化和新农合全市统筹，城乡低保标准进一步提高。保障性安居工程新开工7426套，基本建成9059套，超额完成省定年度任务。优先发展教育事业，梅列、三元区通过义务教育发展基本均衡区国家督导评估。稳步推进医药卫生体制改革，全市22家县级以上医院出院者平均费用4739.98元、下降4%，药品费用比重28.67%、下降11.3个百分点。繁荣文化事业，文化惠民工程扎实开展，万寿岩国家考古遗址公园获立项。“诚信计生·幸福家庭”创建工作全面推进，在全省率先对计生特殊家庭实行全方位救助帮扶，人口计生工作进入全省先进行列。全民健身运动广泛开展，三明市少体校被授予国家高水平体育后备人才基地。推进“平安三明”建设，加强信访工作，开展安全生产大检查和重点整治“百日行动”，强化食品药品安全监管，社会保持安定稳定。青少年、妇女儿童、残疾人工作扎实开展，司法、统计、科普、民族宗教、档案、地方志、水文、气象、防震、库区移民工作取得新进展。深化双拥共建，人民防空、民兵预备役和国防后备力量建设得到加强。

　　（六）在转变职能中提高政府服务水平。坚持依法行政，推进政务公开，自觉接受监督，广泛听取意见，认真办理人大代表建议和政协提案。进一步精简审批事项，启动新一轮市级行政审批项目清理工作，推行项目容缺后补审批，规范投资项目部门备案和中介机构管理。开展行政服务标准化建设，健全市县乡村四级行政服务网络，加快生产性企业“两证”办理。强化政府绩效管理，机关效能建设不断加强。严格落实中央“八项规定”，坚决反对“四风”，开展“四下基层”活动，厉行勤俭节约，深化纠风专项治理，强化行政监察和审计监督，廉政建设和反腐败工作扎实推进。

　　各位代表，过去一年的成绩来之不易，是在省委、省政府和市委正确领导下，在历届政府打下的良好基础上，经过全市上下奋力拼搏取得的。在此，我代表市人民政府，向全市人民，向人大代表、政协委员、各民主党派、工商联、各人民团体、无党派人士、离退休老同志和社会各界人士，向中央和省属驻明单位、驻明部队、武警官兵、公安政法干警，向关心支持三明发展的港澳台同胞、海外侨胞、国际友人，致以衷心的感谢和崇高的敬意！

　　在看到成绩的同时，我们也清醒认识到我市经济社会发展还存在不少困难与问题，主要是：三次产业结构不尽合理，产业实力不强，企业自主创新能力不足，生态保护和污染减排压力较大。发展后劲不够持续，大项目、好项目落地不多。中心城市辐射带动作用不够突出，城市管理有待完善，县域经济实力不强，农村基础设施比较薄弱，城乡居民持续增收难度较大。社会事业发展相对滞后，与群众期盼还有差距。影响社会和谐稳定的因素依然存在。一些干部的宗旨意识、办事效率、服务水平有待提高。对此，我们将高度重视，采取有力措施加以解决。

　　二、2014年主要任务

　　今年是全面贯彻落实党的十八届三中全会、全面深化改革的开局之年。政府工作的总体要求是：以邓小平理论、“三个代表”重要思想、科学发展观为指导，深入贯彻党的十八大和十八届二中、三中全会和习近平总书记系列重要讲话精神，全面落实中央经济工作会议和省委九届十次全会、市委八届七次全会以及省、市经济工作会议部署，坚持稳中求进、改革创新，以全面深化改革为突破口，着力保持经济平稳较快增长，着力提高发展的质量和效益，着力加快产业发展，着力保障和改善民生，着力改善人居环境，全力推进三明科学发展跨越发展，努力实现“百姓富、生态美”有机统一。

　　今年我市经济社会发展主要预期目标是：全市生产总值增长11%；地方公共财政收入增长13%；固定资产投资增长20%；外贸出口增长10%，实际利用外商直接投资增长8%；城镇登记失业率控制在3.8%以内；社会消费品零售总额增长14%，居民消费价格总水平涨幅控制在3.5%左右；人口自然增长率控制在9.5‰以内；城镇居民人均可支配收入增长10%，农民人均纯收入增长10.5%；完成单位生产总值能耗降低和主要污染物减排年度任务。围绕上述目标，着重做好六个方面工作：

　　（一）着力扩大内需，促进经济持续增长

　　实施深化“五大战役”重点行动计划。全力推进产业转型升级、宜居环境建设、城乡基础设施提升、现代农业加快发展、民生工程行动计划，确保完成年度目标任务。强化“三维”项目对接，深化与央企合作发展，重点推进15个在建和前期项目；加强民企项目对接落实，力争全年签约民企合同项目投资额300亿元以上；积极对接大型台港澳侨企业，力争实际利用外资1.2亿美元以上。

　　实施“六大领域”提升工程。交通领域重点加快南三龙铁路建设，推进浦建龙梅、长泉铁路前期工作；抓紧漳永、湄渝高速公路三明莘口至明溪城关段建设，开工建设厦沙高速公路，续建和新开工国省干线185公里，继续推进农村公路建设；加快三明沙县机场建设，力争年内基本建成。能源领域重点抓好三明核电等项目前期工作，加快海西天然气管网三明段一期工程建设，开工一批输变电工程，继续改造提升农村电网。市政领域重点推进城市立面改造、重点路段“白改黑”等项目，加快三明市区第二供水工程建设。水利领域重点抓好防洪、水库、流域治理等方面的23个重大水利项目建设。信息领域重点推进信息网络、公共服务、重点应用、消费服务、信息安全等项目建设。环保领域重点推进省级试点小城镇污水处理厂及配套管网、三明市生活垃圾焚烧发电等项目建设。

　　扩大消费需求。落实促进消费政策，鼓励大众消费，加快发展健身、休闲、文化等新兴消费业态，促进汽车、家电、餐饮等消费。搞活城乡消费市场，加快各类专业市场建设，发展农村超市、社区便利店，培育消费新的增长点。支持限上商贸企业发展，力争年内新增限上商贸企业30家。扶持建筑业企业上等级，推动建筑业加快发展。加强房地产市场调控，增加中小户型普通商品住房供应，促进房地产业平稳健康发展。强化市场监管和价格调控，保障食品药品安全，着力优化消费环境。

　　（二）注重转型升级，促进发展方式转变

　　推进新型工业化。按照国家化解产能过剩要求，打好钢铁、水泥产业结构调整主动仗，做大总量，提升竞争力。落实工业产业链拓展延伸实施方案，着力抓好144个重点项目建设，扶持和培育一批龙头企业，加快冶金及压延、装备制造、林产加工、矿产加工、纺织、生物医药及保健品等六大产业链发展。制定实施信息化与工业化深度融合行动方案，抓好300个投资千万元以上重点技改项目，力争完成技改投资600亿元以上。加快15个重点工业园区开发建设，优化产业功能布局，提高投资强度和产出效益。落实省上进一步推动工业稳增长促转型11条措施，加大中小微企业扶持力度，帮助企业解决融资、用地、用工等问题。制定实施矿产资源整合方案，加快引进战略投资者，推动资源往产业集聚发展。

　　提升现代服务业。大力发展金融、信息、会展、现代物流、服务外包等生产性服务业，加快发展家政、养老、社区服务等生活性服务业，积极发展连锁经营、物流配送等现代商贸业态。推进电子商务发展，重点抓好三明林业大宗商品交易中心、闽光现代物流、清流春舞枝花卉电子交易中心、永安电商大厦等项目建设，培育壮大一批电子商务龙头企业。加快旅游业发展，推进市区旅游项目开发，支持泰宁县旅游综合体制改革，整合旅游资源，培育市场主体，推出精品线路，完善配套服务，加强旅游营销，拓展旅游链条，做大动车旅游经济。

　　强化科技和人才支撑。健全技术创新市场导向机制，突出企业创新主体地位，鼓励企业增加研发投入，推进企业技术、产品、品牌创新，加强知识产权运用和保护。推进产学研协同创新，加快科技平台建设，支持机械科学研究总院海西分院等研发龙头发展，深化与清华大学等高校和科研院所合作，实施70个以上重大科技项目，新建30家以上企业研发机构，建成8家以上科技企业孵化器，新增3家以上省级院士专家工作站，创建3家以上省级博士后创新实践基地，力争高新技术产业产值增长12%以上。注重人才培养和引进，落实和完善人才集聚政策，优化人才发展环境，吸引更多人才来明留明就业创业。

　　（三）突出“三农”工作，促进农民增收致富

　　切实保障粮食安全。完善粮食生产扶持政策，确保粮食播种面积稳定在320万亩、粮食总产稳定在110万吨。提升粮食安全保障能力，加强农田水利和高标准农田建设，实施农业综合开发土地整理15.2万亩，确保耕地占补平衡。加强动植物疫病防控，建立原产地可追溯制度和质量标识制度，确保农业生产和农产品质量安全。加强副食品生产基地建设，推进农超对接，确保市场供应充足、价格基本稳定。

　　大力发展现代农业。培育特色规模，重点围绕烟叶、茶叶、种子、优质大米、莲子、油茶、食用菌等特色农产品，加大扶持力度，打响产品品牌，壮大产业规模。推进农业产业化经营，加快现代农业示范区、台湾农民创业园、福建农民创业园和农民创业示范基地建设，大力发展设施农业、生态农业、观光农业，引导农业向加工、流通环节延伸。构建新型农业经营体系，培育壮大农业经营主体，做大做强农业龙头企业，规范提升农民专业合作社，鼓励发展家庭农场。强化农业科技创新，开展农业“五新”成果对接，推进现代种业发展，加大良种的引进、繁育与推广力度。抓好基层农业公共服务机构能力建设，提升农业社会化服务水平。

　　改善农村生产生活条件。扎实推进美丽乡村创建活动，每个县（市、区）抓好1个美丽乡镇试点，全市开展100个以上美丽村试点。延伸拓展“点线面”攻坚，继续抓好农村环境综合整治，重点推进向莆铁路、高速公路沿线环境整治，进一步改善城乡面貌。实施造福工程搬迁和危房改造4500户以上，解决10万农村人口的饮水安全问题。用好苏区老区政策，继续实施老区村跨越发展工程。支持少数民族乡村发展，做好库区移民安置和后期扶持工作。

　　提高农民收入水平。拓宽农民增收渠道，落实强农惠农富农政策，积极发展农民股份合作，加强农民技能培训，大力培养新型农民，增强就业创业能力，提高中等收入农民比重。深入推进扶贫开发，落实省上支持扶贫开发工作重点县加快发展政策，实施第四轮整村推进扶贫开发工作，鼓励发展农产品深加工、林下经济、休闲农业、生态旅游等特色经济，增强贫困地区自我发展能力。

　　（四）加快城乡建设，促进城镇化健康发展

　　推进中心城市建设。大力发展市区经济，强化产业建设，支持三钢等龙头企业发展，培育新兴业态，增强城市辐射带动力。加快交通基础设施建设，推进205国道、306省道改线工程，年内建成三明中心城市快速通道。抓紧市区重点地块土地收储出让工作，加快徐碧新城二期、贵溪洋、台江、东霞-富兴堡等重点板块开发。加强和创新城市管理，强化城区薄弱区域基础设施建设，清理整治违法占地、违法建设，进一步提升城市形象。加快市区城市绿道项目建设，继续实施市区沙溪两岸绿地景观改造工程，抓好迎接国家园林城市复检工作。

　　提升生态工贸区建设水平。按照“十同”要求，继续加快三明市区与沙县、永安同城化。进一步落实生态工贸区发展规划和促进产业发展的若干意见，加快生态新城开发建设，编制完成生态新城核心区控制性规划及各专项规划，明确“一带六区”功能定位，全面推进道路、管网、绿化等配套设施建设。强化产城联动，加快引进一批重大产业项目，推动“陆地港”二期等项目建设，大力发展符合生态新城产业布局、人口聚集、生活配套的第三产业。

　　加快县域经济发展。抓好县城规划和建设，有序推进新区开发和旧城改造，完善配套公共设施，提升县城特色与品位。加强县城与园区联动，推动以城促产、以产兴城，打造人流、物流、资金流集聚的县域中心。制定实施我市新型城镇化规划，继续加快4个省级试点镇和16个市级中心乡镇建设，科学稳妥推进强镇扩权，抓好小城镇行政管理体制、土地经营机制等改革，逐步建立多元可持续的资金保障机制。有序推进农业转移人口市民化，放开进城落户限制，推动农业转移人口逐步享受与当地城镇居民同等的公共服务和社会福利，增强小城镇集聚功能，提高城镇化质量。

　　加强生态文明建设。加快国家级生态市和国家环保模范城市创建工作，力争年内达到国家环保模范城市考核指标规定要求，6个县通过国家级生态县技术评估，80%的乡镇达到国家级生态乡镇标准，初步建成省级生态市。推进南方林区生态文明建设综合配套改革实验区试点，完善生态保护红线划定工作，强化重要生态功能区保护，推进矿山生态修复，抓好造林绿化和“四绿”工程，扎实创建国家森林城市。加强水土保持工作，实施5个国家重点县和4个省级重点县治理项目，综合治理水土流失面积30万亩。严格落实节能减排目标责任和环境保护“一岗双责”，实施重点节能技术改造项目30项、循环经济项目20项，全面推进污染减排，抓好脱硫脱硝、废水深度治理等一批重点减排项目。加强重点流域水环境综合治理，强化湖泊生态环境保护。推进国家餐厨废弃物资源化利用和无害化处理试点城市工作。

　　（五）深化改革开放，促进体制机制创新

　　推进重点领域改革。稳定农村土地承包关系，用活土地经营权，推广农民以承包经营权入股、承包经营权信托、土地托管等流转方式，推动土地适度规模经营，加快永安国家级土地承包经营权确权登记颁证试点工作。完善集体林权制度改革，构建新型林业经营体系，搭建森林资源规范流转平台，稳妥推进林权资本化，提升林业经营实效，建立集体林区良性发展机制。深化金融改革，推广沙县农村金融改革经验，积极引进区外银行在我市设立分支机构，促进基金投资、资产管理公司等在我市落户发展。推动企业上市融资，力争1-2家企业实现上市，支持企业发行公司债券、中小企业区域集优票据，提高直接融资比重。深化财税体制改革，稳步推进财政预决算公开，加快构建现代财政制度，着力控制地方政府性债务风险。推动国有企业和国资监管体制改革，做大做强市城投公司、国投公司、省一建公司、交投公司等国有企业，积极发展混合所有制经济。加快总部经济发展，力争新引进总部经济企业6家以上。稳步实施政府机构改革，抓好事业单位分类改革。创新市、区行政管理体制，认真落实城市管理相对集中行政处罚权工作。推进工商登记制度改革。

　　扩大对外合作。创新招商引资方式，突出产业链招商，强化招大商招好商，优化招商服务，推动外商投资便利化。落实扶持外贸出口发展政策，支持生产型外贸企业自营出口，扩大地产品出口。拓展明台交流合作，加快海峡两岸现代林业合作实验区、三明台商投资区建设，办好第十届林博会。深化区域协作，推动与沿海共建产业园。加强与港澳地区、国外友城的沟通联系，做好与海外华侨华人及其社团的联络工作。

　　加快民营经济发展。落实支持民营经济发展的政策措施，推动民间资本健康发展，鼓励和引导民间资本以多种方式进入基础产业、社会事业以及特许经营领域，继续抓好生产性企业“两证”办理，进一步改善民营经济发展环境。实施“回归工程”，发展壮大商会经济。支持民营企业做大做强，力争新增产值超亿元企业25家、税收超百万元企业50家。

　　（六）强化民生保障，促进发展成果共享

　　加强就业和社会保障。实施更加积极的就业政策，促进以高校毕业生为重点的青年就业和农村转移劳动力、城镇困难人员、退役军人就业，全年新增城镇就业2.86万人、农村富余劳动力转移就业4.5万人。稳步推进机关事业单位收入分配制度改革。提高城乡居民养老保险基础养老金标准。加快社会养老服务体系建设，全面完成县级福利中心和乡镇敬老院建设任务。深化医药卫生体制改革，继续完善市县公立医院综合改革，完善院长年薪制和医生（技师）年薪制，适时推行县级以上医院法人治理结构改革，抓好30个常见病单病种付费改革，推进乡镇卫生院新一轮改革。提高新农合保障水平，鼓励引导社会资本办医，加强重大疾病防控和卫生监督。加快公共租赁住房、廉租住房等保障性住房建设，新建保障性住房和改造棚户区4500套以上，做好分配和管理工作。落实低保标准与最低工资标准挂钩的调整机制、与物价上涨挂钩的联动机制，提高城乡低保水平。改善优抚对象待遇，加强残疾人权益保障，加快发展社会福利、红十字会、慈善、老龄事业，保障妇女、儿童合法权益。

　　加快社会事业发展。深化教育领域综合改革，实施新一轮学前教育三年行动计划，支持民办幼儿园发展，实现农村学前教育全覆盖。提升义务教育学校标准化建设水平，加快城区中小学扩容工程建设，抓好农村中小学生“幸福成长工程”，推进普通高中多样化发展，扩大优质教育资源覆盖面，促进教育公平。积极推进义务教育学校校长教师校际交流。稳妥推进考试招生制度改革。整合职业教育培训资源，推进职业教育集团化办学，创建国家示范性高职院校。落实高校办学自主权，推进开放办学，提高办学水平和人才培养质量。鼓励和支持社会力量兴办教育，加快特殊教育、民族教育、社区教育、老年教育发展。抓好市中小学生示范性综合实践基地、职教园区等项目建设。坚持计划生育基本国策，启动实施一方是独生子女的夫妇可生育两个孩子政策，深入开展创建幸福家庭全国试点市工作，加强生育关怀，促进人口计生工作持续健康发展。继续深入开展全民健身运动，培养竞技体育后备人才，加快市少体校新校区建设，积极备战省第十五届运动会。做好科普工作，提高全民科学素质。加强统计工作，抓好民族宗教、档案、地方志、水文、气象、防震等工作。

　　推进文化事业和文化产业发展。培育和践行社会主义核心价值观，抓好国家公共文化服务体系示范区创建工作，继续实施文化惠民工程，基本建成市新图书馆、艺术馆、青少年宫等项目，完成市科技馆布展工作，强化基层文化服务阵地和设施建设，促进基本公共文化服务均等化。加强文物和非物质文化遗产保护，加快建设万寿岩国家考古遗址公园。深化文化体制改革，抓好市级广电机构管理运营机制创新，整合文化广电经营性资产，探索建立文化产业融资担保公司。按照“一县一品牌、一县一园区、一县一实体”的要求，加快文化旅游、文化创意、新闻出版、广播影视等文化产业发展，培育文化产业龙头企业和重点园区，打造客家文化等区域文化品牌，增强文化产业实力。繁荣社会科学，发展文学艺术，创作文化精品。

　　创新社会治理。做好迎接全国文明城市总评工作，巩固全国未成年人思想道德建设工作先进城市成果。改进社会治理方式，深化“平安三明”创建活动，完善立体化社会治安防控体系，健全重大决策社会稳定风险评估机制，依法依规处理信访事项，有效化解社会矛盾。提升各类社会组织承接政府职能转移能力，推进重点行业和领域信用体系建设。严格落实安全生产责任，建立隐患排查治理体系和安全预防控制体系，推进安全生产标准化建设，继续开展道路交通安全综合整治“三年行动”和“清剿火患”战役，遏制重特大安全事故。健全防灾减灾体系，提升应急处置能力。推进社区网格化服务管理，强化社区矫正工作。抓好法制宣传教育，完善法律援助制度。发展基层民主，畅通民主渠道，完善村（居）民自治。加强与各民主党派、工商联、无党派代表人士的联系与协商，支持工会、共青团、妇联等人民团体依照法律和各自章程开展工作。抓好国防动员、人民防空、民兵预备役、双拥共建工作，推进军民融合深度发展。

　　三、切实加强政府自身建设

　　（一）求真务实改作风。认真落实中央“八项规定”和省委、市委要求，深入开展党的群众路线教育实践活动和“四下基层”活动，聚焦“四风”找问题、抓整改，养成良好作风。加强调查研究，直奔生产一线、直达项目现场、直击困难矛盾，在基层接触地气、摸清实情，在一线靠前协调、解决问题。改进文风会风，减少会议、文件，减少一般性考核、检查、评比活动，把时间和精力用在为民服务和推动工作上。

　　（二）强化服务转职能。做好简政放权文章，把中央和省上明令取消的、政府不该管的，不折不扣地放给市场、交给社会，一律取消不符合法律规定、利用红头文件设定的管理、收费、罚款项目，更多释放市场活力，以权力的“减法”换发展的“乘法”。进一步减少审批事项、精简审批环节，推进审批服务标准化管理，落实容缺后补审批制度，加强投资项目部门备案和中介机构的规范化管理，更好地服务群众、方便群众。加强行政服务中心建设，提升窗口服务质量，提高部门及项目入驻率。推进绩效管理规范化，抓好机关效能建设，落实“马上就办、办就办好”，着力治庸治懒治散，防止政策打折扣、办事中梗阻、效益层层递减。

　　（三）雷厉风行抓落实。对中央、省和市委作出的决策部署，坚决从速从快贯彻执行到位，特别是对国家和省上出台的各项政策措施，抓住“第一时间”学习研究，做到“第一反应”生成项目，抢出“第一效益”赢得发展。对市政府议定的事项、作出的承诺，立说立行、只争朝夕，快节奏推进、高效率完成，切实做到言必信、行必果。对看准的事项、能拍板的工作，各级政府各部门要敢于担当、果断行事，乘势而不等示、进取而不等待、主动而不被动，以最快的速度把工作落到实处。

　　（四）公开透明促廉洁。自觉接受人大及其常委会的法律、工作监督和政协的民主监督，优质高效办理建议提案，主动接受社会公众和新闻舆论监督。推进法治三明建设，坚持依法科学民主决策，深化政务公开和政府信息公开，加强行政复议和行政应诉，严格规范公正文明执法，使行政在法治轨道上有力推进。严格执行《党政机关厉行节约反对浪费条例》和《党政机关国内公务接待管理规定》，严控一般性支出，严控机构编制和财政供养人员总量，严控“三公”经费。落实党风廉政建设责任制，推进源头治理、制度防腐，进一步加强和规范政府投资项目招投标管理，强化对工程建设、土地出让、矿产资源配置、产权交易和政府采购等关键领域的廉政风险防控，严肃查处违法违纪案件，营造风清气正的政务环境。

　　各位代表，改革催人奋进，发展任重道远。让我们紧密团结在以习近平同志为总书记的党中央周围，在市委的正确领导下，紧紧团结和依靠全市人民，振奋精神，开拓创新，扎实进取，为推动三明科学发展跨越发展、加快全面建成小康社会而努力奋斗！
